# Risk Factors for Pseudomyxoma Peritonei in Appendiceal Mucinous Neoplasms: A Single-Center Experience

**DOI:** 10.7759/cureus.104838

**Published:** 2026-03-07

**Authors:** Jorge Luis Leal-Hidalgo, Kevin Joseph Fuentes-Calvo, Sara Fernanda Arechavala-Lopez, Irving Fuentes-Calvo, Luis Felipe Arias-Ruiz, Rita Dorantes-Heredia, Mauricio Gutierrez-Alvarez, Artemio Garcia-Badiola, Daniel Camacho-Mauries, Eduardo Esteban Montalvo-Jave

**Affiliations:** 1 General Surgery, Facultad Mexicana de Medicina, Universidad La Salle México, Mexico City, MEX; 2 General Surgery, Hospital Médica Sur, Mexico City, MEX; 3 Medicine, Hospital Médica Sur, Mexico City, MEX; 4 Anatomic Pathology, Hospital Médica Sur, Mexico City, MEX; 5 Plastic and Reconstructive Surgery, Hospital Central Sur Petróleos Mexicanos (PEMEX), Mexico City, MEX; 6 Colon and Rectal Surgery, Hospital Médica Sur, Mexico City, MEX; 7 General Surgery, Facultad de Medicina, Universidad Nacional Autónoma de México (UNAM), Mexico City, MEX

**Keywords:** appendiceal mucinous neoplasm, appendiceal perforation, oncologic surgery, pseudomyxoma peritonei, risk factors

## Abstract

Introduction: Appendiceal mucinous neoplasms (AMNs) are rare tumors with the potential to progress to pseudomyxoma peritonei (PMP). Identification of prognostic factors may improve surgical management and follow-up strategies.

Objective: This study aimed to analyze clinicopathological factors associated with the development of PMP in patients with AMNs treated at a tertiary care hospital in Mexico.

Methods: A retrospective study was conducted in Hospital Médica Sur, Mexico City, Mexico, including 30 patients with histologically confirmed AMNs between 2018 and 2023. Clinical, surgical, histological, and laboratory variables were analyzed. Bivariate analyses and binary logistic regression models were performed to identify factors associated with the development of PMP.

Results: Appendiceal perforation was the only factor significantly associated with the development of PMP, as the absence of perforation was linked to significantly lower odds of disease (odds ratio (OR): 0.090; 95% confidence interval (CI): 0.010-0.798; p = 0.031). Histological subtypes other than low-grade appendiceal mucinous neoplasm (LAMN) showed a nonsignificant trend toward association (p = 0.076).

Conclusion: Appendiceal perforation was the main factor associated with the development of PMP in this cohort. Its identification may help guide surgical decision-making and individualized postoperative surveillance strategies.

## Introduction

Appendiceal mucinous neoplasms (AMNs) represent a rare group of appendiceal tumors, with an estimated incidence ranging from 0.2% to 0.7% in appendectomy specimens [1,2]. These lesions encompass a histopathological spectrum that ranges from low-grade appendiceal mucinous neoplasms (LAMNs) to mucinous adenocarcinomas with high malignant potential [3]. Their clinical presentation is often nonspecific, frequently mimicking acute appendicitis or pelvic masses, which complicates preoperative diagnosis [2,4].

The term “appendiceal mucocele” was first described by Rokitansky in 1842 to refer to appendiceal distension caused by mucin accumulation, regardless of its underlying etiology [5]. Currently, it is considered a nonspecific morphological finding rather than a definitive diagnosis [3]. For appropriate classification, the Peritoneal Surface Oncology Group International (PSOGI) proposed in 2016 a standardized categorization of AMNs into low-grade (LAMN), high-grade (HAMN), and mucinous adenocarcinomas [6]. This classification has gained clinical relevance because histological subtypes exhibit distinct biological behavior: while LAMNs generally have a low potential for dissemination when resected intact, HAMNs and mucinous adenocarcinomas demonstrate greater aggressiveness, a higher risk of rupture, and increased rates of intraperitoneal recurrence [6,7]. Histology, therefore, not only informs prognosis but may also guide surgical decision-making and postoperative surveillance.

One of the most severe complications of AMNs is the development of pseudomyxoma peritonei (PMP), an entity characterized by the dissemination of mucin and neoplastic epithelial cells throughout the peritoneal cavity [8]. The risk of progression to PMP has been associated with factors such as appendiceal rupture, the presence of extracellular mucin, and positive surgical margins [9]. However, evidence regarding clinicopathological predictors of PMP remains limited, particularly in Latin American populations.

Given the variable biological behavior of these lesions and their potential to progress to PMP, identifying factors associated with this progression is essential. The aim of this study is to analyze clinicopathological risk factors in patients with AMNs treated at a tertiary care center in Mexico and to explore their association with the development of PMP.

## Materials and methods

A retrospective observational study was conducted in Hospital Médica Sur, Mexico City, Mexico, including 30 patients with histologically confirmed AMNs treated between 2018 and 2023 at a tertiary care center. Patients operated on by the general surgery department were included if they had complete medical records and a minimum follow-up of six months to assess the presence or development of PMP. Patients without pathological confirmation of mucinous neoplasia, those with a prior diagnosis of PMP, or those with a concomitant malignant neoplasm were excluded. Additional surgical procedures, such as right hemicolectomy or hyperthermic intraperitoneal chemotherapy (HIPEC), were recorded as clinical variables when performed.

Data collection was performed through a review of medical records and institutional databases and was recorded using the most recent available version of Microsoft Excel for subsequent analysis. Demographic, surgical, and histopathological variables were collected, including age, sex, type of surgery, histological subtype, appendiceal perforation, surgical margins, mucin deposits, HIPEC, complicated appendicitis, and tumor diameter assessed by imaging. Laboratory variables included hemoglobin, leukocyte count, C-reactive protein, CA 19-9, CA 125, and carcinoembryonic antigen (CEA). The dependent variable was the presence or subsequent development of PMP, identified either at the time of diagnosis or during follow-up. Appendiceal perforation was defined as any documented disruption of the appendiceal wall identified intraoperatively or reported in the pathology report.

Descriptive analyses were performed using absolute and relative frequencies for categorical variables and means with standard deviations for continuous variables. Normality of continuous variables was assessed using the Shapiro-Wilk test. Normally distributed variables were compared using the Student’s t-test, whereas non-normally distributed variables were analyzed using the Mann-Whitney U test. Categorical variables were analyzed using the chi-square test or Fisher’s exact test, as appropriate. A p-value < 0.05 was considered statistically significant. Given the limited number of outcome events, multivariate analyses were interpreted cautiously and considered exploratory.

A binary logistic regression model was constructed to identify clinicopathological factors associated with the development of PMP. Variables included in the model were selected based on clinical relevance, prior evidence in the literature, and their statistical behavior in the bivariate analysis. The model included appendiceal perforation and histological subtype recoded as LAMN versus non-LAMN. Variables were entered simultaneously using the “Enter” method. Adjusted odds ratios (ORs) with corresponding 95% confidence intervals were calculated. Model goodness-of-fit was assessed using the Hosmer-Lemeshow test. Given the limited sample size and the small number of outcome events, this analysis was considered exploratory. All statistical analyses were performed using IBM SPSS Statistics for Windows, version 29.0 (released 2022, IBM Corp., Armonk, NY).

The study was approved by the Research Ethics Committee of Hospital Médica Sur (Comité de Ética en Investigación de Médica Sur) (protocol number: 2024-EXT-848).

## Results

Clinical and sociodemographic characteristics

Among the 30 patients with a histopathological diagnosis of AMN, the mean age was 60.6 ± 15.3 years (range: 15-85). The 25th, 50th, and 75th percentiles for age were 53.5, 64, and 73 years, respectively. In this cohort, 20 patients (66.7%) were female and 10 (33.3%) were male (Table 1).

**Table 1:** Descriptive analysis of the study population (n = 30). Data are presented as number (%), mean ± SD. LAMN: low-grade appendicular mucinous neoplasm, HAMN: high-grade appendicular mucinous neoplasm, G1: Grade 1, G2: Grade 2, HIPEC: hyperthermic intraperitoneal chemotherapy, CRP: C-reactive protein, CEA: carcinoembryonic antigen

Variable		n = 30
Age (years)		60.6 (±15.3)
Sex (%)	Men	10 (33.3%)
	Women	20 (66.7%)
Histological type	LAMN	20 (66.7%)
	HAMN	8 (26.7%)
	G1	1 (3.3%)
	G2	1 (3.3%)
Appendicular perforation (%)		9 (30.0%)
Peritoneal pseudomyxoma (%)		8 (26.7%)
Positive surgical margin (%)		1 (3.3%)
Type of surgery (%)	Laparoscopic	15 (50.0%)
	Open	14 (46.7%)
HIPEC performed (%)		7 (23.3%)
Mucin deposits (%)		8 (26.7%)
Complicated appendicitis (%)		13 (43.3%)
Tumor diameter by image (mm)		35.7 (±44.4)
Hemoglobin (g/dL)		13. 5 (±2.1)
Leukocytes (×10³/μL)		8.5 (±3.5)
CRP (mg/L)		42.0 (±33.4)
CA 19-9 (U/mL)		43.6 (±70.1)
CA 125 (U/mL)		124.7 (±221.5)
CEA (ng/mL)		22.6 (±27.6)

Histopathological findings

Regarding histological subtype, 20 patients (66.7%) were diagnosed with LAMN, eight (26.7%) with HAMN, and one case (3.3%) each corresponded to G1 and G2 subtypes. Appendiceal perforation was observed in nine cases (30%), and PMP was identified in eight patients (26.7%) in this cohort.

Perioperative findings

Positive surgical margins were identified in one patient (3.3%). The surgical approach was laparoscopic in 15 patients (50.0%) and open in 14 patients (46.7%); the surgical approach data were unavailable for one patient. HIPEC was performed in seven patients (23.3%). Mucin deposits were identified in eight cases (26.7%), and complicated appendicitis was observed in 13 cases (43.3%).

The mean tumor diameter measured by imaging was 35.7 ± 44.4 mm. Laboratory findings showed a mean hemoglobin level of 13.5 ± 2.1 g/dL, leukocyte count of 8.5 ± 3.5 × 10³/μL, and C-reactive protein (CRP) level of 42.0 ± 33.4 mg/L. Mean tumor marker values were 43.6 ± 70.1 U/mL for CA 19-9, 124.7 ± 221.5 U/mL for CA 125, and 22.6 ± 27.6 ng/mL for carcinoembryonic antigen (CEA).

Statistical analysis

Clinical, surgical, and laboratory characteristics were compared between patients diagnosed with PMP (n = 8) and those without this complication (n = 22) (Table 2). No significant differences were observed in age (60.8 ± 11.7 vs. 60.5 ± 16.6 years; p = 0.965) or sex distribution (p = 1.000).

**Table 2:** Comparison of clinical characteristics between patients with and without peritoneal pseudomyxoma. Data are presented as number (%), mean ± SD. Values in bold indicate P < 0.05. P-values were calculated using Student’s t test or Mann–Whitney U test for continuous variables, and chi-square test for categorical variables, as appropriate. For nonparametric comparisons, the Mann–Whitney U test was used, and Z values are reported as test statistics. χ²: chi-square test, t: Student’s t test, LAMN: low-grade appendicular mucinous neoplasm, HAMN: high-grade appendicular mucinous neoplasm, G1: Grade 1, G2: Grade 2, PMP: peritoneal pseudomyxoma, HIPEC: hyperthermic intraperitoneal chemotherapy, CRP: C-reactive protein, CEA: carcinoembryonic antigen

Variable		PMP positive (n = 8)	PMP negative (n = 22)	Test statistic	P-value
Demographics					
Age (years)		60.8 (±11.7)	60.5 (±16.6)	t = −0.044	0.965
Sex (%)	Men	3 (37.5%)	7 (31.8%)	χ² = 0.085	0.770
	Women	5 (62.5%)	15 (68.2%)		
Intraoperative data					
Histological type	LAMN	2 (25.0%)	18 (81.8%)	χ² = 13.125	0.004
	HAMN	6 (75.0%)	2 (9.1%)		
	G1	0 (0%)	1 (4.5%)		
	G2	0 (0%)	1 (4.5%)		
Appendicular perforation (%)		6 (75.0%)	3 (13.6%)	χ² = 10.519	0.001
Positive surgical margin (%)		0 (0%)	1 (4.5%)	χ² = 0.376	0.540
Type of surgery (%)	Laparoscopic	5 (62.5%)	10 (47.6%)	χ² = 0.514	0.474
	Open	3 (37.5%)	11 (52.4%)		
HIPEC performed (%)		3 (37.5%)	4 (19.0%)	χ² = 1.077	0.299
Mucin deposits (%)		6 (75.0%)	2 (9.1%)	χ² = 13.032	0.001
Complicated appendicitis (%)		5 (62.5%)	8 (38.1%)	χ² = 1.395	0.238
Tumor diameter by image (mm)		21.3 (±14.9)	40.7 (±50.3)	t = 0.918	0.369
Laboratory values					
Hemoglobin (g/dL)		13.9 (±1.9)	13.3 (±2.2)	t = −0.571	0.638
Leukocytes (×10³/μL)		6.5 (±1.2)	9.2 (±3.8)	Z = 2.10	0.036
CRP (mg/L)		3.9 (±11.4)	51.5 (±29.7)	t = 1.434	0.247
CA 19-9 (U/mL)		21.8 (±20.1)	50.9 (±84.1)	Z = 0.00	1.000
CA 125 (U/mL)		50.9 (±25.2)	161.6 (±275.9)	t = 0.534	0.621
CEA (ng/mL)		20.0 (±4.4)	24.4 (±38.7)	Z = -1.75	0.080

Among intraoperative findings, histological subtype showed a statistically significant difference (p = 0.004), with a higher proportion of HAMN in the PMP group (75.0% vs. 9.1%). Appendiceal perforation was also significantly more frequent in patients with PMP (75.0% vs. 13.6%; p = 0.001). Similarly, mucin deposits were more commonly observed in the PMP group (75.0% vs. 9.1%; p = 0.001).

No significant differences were found regarding surgical approach (p = 0.682), use of HIPEC (p = 0.357), or presence of positive surgical margins (p = 1.000). In laboratory analyses, leukocyte levels were significantly lower in the PMP group (6.5 ± 1.2 vs. 9.2 ± 3.8 × 10³/μL; p = 0.036). Hemoglobin, CRP, CA 19-9, CA 125, and CEA levels did not show statistically significant differences. Tumor diameter was also smaller in the PMP group, without reaching statistical significance (21.3 ± 14.9 vs. 40.7 ± 50.3 mm; p = 0.075).

Clinical-surgical analysis

Binary logistic regression analysis suggested that appendiceal perforation was independently associated with the development of PMP. The adjusted odds ratio was 0.090 (95% CI: 0.010-0.798; p = 0.031), indicating that the absence of appendiceal perforation was associated with significantly lower odds of developing PMP (Table 3).

**Table 3:** Multivariate analysis of binary logistic regression for clinical-surgical factors associated with the development of peritoneal pseudomyxoma. Values in bold indicate P < 0.05. Statistical significance in logistic regression models was assessed using the Wald chi-square test. OR: odds ratio, CI: confidence interval, LAMN: low-grade appendicular mucinous neoplasm

Variable	OR	95% CI	Wald χ²	P-value
Absence of appendicular perforation	0.090	0.010–0.798	4.679	0.031
Histological type (Non-LAMN)	0.139	0.016–1.228	3.152	0.076

Histological subtype (non-LAMN vs LAMN) was not independently associated with the development of PMP (OR: 0.139; 95% CI: 0.016-1.228; p = 0.076). 

These findings suggest that intraoperative appendiceal perforation may represent a clinically relevant risk factor for progression to PMP. However, these findings should be interpreted cautiously, given the limited sample size and number of outcome events.

## Discussion

This study identified clinicopathological factors associated with the development of PMP in patients with AMNs. Multivariate analysis suggested that appendiceal perforation was associated with the development of PMP, whereas histological subtypes other than LAMN showed a nonsignificant trend toward association. These findings highlight the potential role of intraoperative factors in the progression of AMNs to PMP.

The frequency of PMP in our cohort (26.7%) is consistent with previously reported data, in which this complication occurs in approximately 20-30% of patients with AMNs, depending on histological grade and appendiceal integrity. Aleter A et al. reported that both tumor subtype and intraoperative findings significantly influence progression to PMP [10], a finding that was also reflected in our bivariate analysis.

Although histological subtypes other than LAMN did not reach statistical significance in the multivariate analysis, a higher proportion of PMP cases corresponded to HAMNs (75%) in our cohort. This observation is consistent with findings reported by Kong et al., who demonstrated that high-grade AMNs exhibit greater aggressiveness and an increased risk of peritoneal dissemination [11]. Similarly, Panarelli and Yantiss described that HAMNs and mucinous adenocarcinomas display a more invasive biological behavior compared with LAMNs [12]. The limited sample size of our cohort may have reduced the statistical power to detect a significant association.

In our study, serum carcinoembryonic antigen (CEA) concentration was not significantly associated with the development of PMP. This finding contrasts with reports such as that by Wang et al., who demonstrated that elevated CEA and CA 19-9 levels correlate with more advanced disease and greater tumor burden [13]. However, studies such as that by Tiselius et al. have also emphasized that tumor markers may be variable and nonspecific, particularly in early-stage disease [14]. Therefore, their utility as predictive tools in small cohorts remains limited.

The findings of this study reinforce the importance of careful assessment of intraoperative factors when making therapeutic decisions. As reported by Soucisse et al., patients with appendiceal rupture or free mucin may exhibit poorer prognosis and may benefit from closer surveillance or consideration of adjuvant strategies such as HIPEC [15]. Early recognition of these factors may enable a more individualized approach, which could potentially improve recurrence-free survival.

This study contributes evidence regarding clinical and surgical factors that may influence the progression of AMNs, with particular emphasis on appendiceal perforation. The findings should be interpreted as exploratory, given the limited sample size and number of events. Nevertheless, they highlight the potential relevance of intraoperative factors in the progression of AMNs to PMP. However, the limited sample size restricts the generalizability of the results and reduces the statistical power to detect less robust associations. Prospective, multicenter studies are needed to validate these findings in larger populations.

Appendiceal perforation was identified as an independent predictor of PMP development, with a statistically significant adjusted odds ratio. This finding is consistent with reports by Honoré et al., who emphasized that appendiceal rupture facilitates mucinous dissemination and peritoneal seeding [16]. It is also demonstrated that, even in low-grade tumors, the presence of perforation significantly increases the risk of recurrence and progression to PMP [16]. These data reinforce the recommendation to avoid rupture during surgical resection and to consider perforation as a high-risk criterion that may modify postoperative management.

## Conclusions

Appendiceal perforation was the only factor significantly associated with the development of PMP in patients with AMNs in this cohort. Although histological subtypes other than LAMN showed a trend toward greater aggressiveness, this association did not reach statistical significance. These findings suggest that intraoperative factors may play an important role in the progression of AMNs to PMP. However, given the limited sample size and number of outcome events, these results should be interpreted as exploratory. Larger studies with greater statistical power are required to confirm these observations and improve risk stratification in this rare but potentially severe condition.
